# Aberrant expression of serum circANRIL and hsa_circ_0123996 in children with Kawasaki disease

**DOI:** 10.1002/jcla.22874

**Published:** 2019-03-06

**Authors:** Junhua Wu, Qianqin Zhou, Yadan Niu, Jiayi Chen, Yingchao Zhu, Shazhou Ye, Yang Xi, Fuyan Wang, Haiyan Qiu, Shizhong Bu

**Affiliations:** ^1^ The Ningbo Women and Children’s Hospital Ningbo China; ^2^ Diabetes Research Center, School of Medicine Ningbo University Ningbo China

**Keywords:** circular non‐coding RNA, intravenous immunoglobulin, Kawasaki disease

## Abstract

**Background:**

Kawasaki disease is a childhood systemic vasculitis that causes coronary artery abnormalities. The etiology remains unknown and there are no specific diagnostic tests. Circular non‐coding RNAs are a special class of endogenous RNAs that display some characteristics of an ideal biomarker. However, few studies have examined the expression of circRNAs in the serum of Kawasaki disease (KD) patients. The aim of this study was to identify circRNAs in the serum that can serve as potential biomarkers for KD diagnosis.

**Methods:**

The cases were children diagnosed with KD (n = 56). The controls comprised healthy children (n = 56). Blood was collected from the patients before and after intravenous immunoglobulin therapy, and from the healthy controls. Levels of circANRIL and hsa_circ_0123996 in the serum were measured by quantitative reverse transcription PCR. Then, the potential relationship between serum circRNA levels and patients’ biochemical parameter levels was investigated. Receiver operating characteristic curves were constructed for evaluating the diagnostic value of these circRNAs.

**Results:**

The serum levels of circANRIL were lower in patients with KD before therapy than in the controls, but became higher in the patients after therapy than before therapy. The serum levels of hsa_circ_0123996 were higher in patients with KD before therapy than in healthy controls.

**Conclusion:**

Our study indicated that the circANRIL and hsa_circ_0123996 levels in the serum of patients with KD were significantly different from those in healthy individuals. circANRIL and hsa_circ_0123996 may become potential biomarkers for early KD diagnosis.

## 1 INTRODUCTION

Kawasaki disease (KD) is a self‐limited childhood systemic vasculitis that affects small and medium‐sized vessels. If left untreated, it results in coronary artery abnormalities in up to 25% of children.^1^ However, the etiology of KD is still unclear. The diagnosis of KD is based solely on clinical features, because there is no specific laboratory test for the early identification and diagnosis of KD.^2^ In an important way, a delay in accurate diagnosis may lead to increased mortality and morbidity from complications of KD.^3^ Thus, improving the diagnosis and treatment of KD is necessary for reducing mortality and morbidity associated with KD.

Circular non‐coding RNAs (circRNAs) are a special class of endogenous RNAs characterized by jointed 3′ and 5′ ends via exon or intron circularization and are broadly expressed in eukaryotic cells, but their involvement in human disease remains to be elucidated.^4^, ^5^ Previously, circRNAs were thought to be expressed at such low levels that they were generally regarded as byproducts of splicing errors and were not considered biologically active. However, the use of next generation sequencing has shown that circRNAs are expressed abundantly and are ubiquitous among eukaryotes.^6^, ^7^ Several circRNAs have been reported to have physiological functions.^8^, ^9^ Additionally, circRNAs have been suggested to be potential novel biomarkers of disease, including cancer,^10^ heart failure,^11^ and Parkinson's disease.^12^ However, there has been no report on the serum circRNAs levels in patients with KD.

Here, we studied circANRIL and hsa_circ_0123996 in KD because they are circRNAs that may be associated with coronary artery and heart failure according to a bioinformatics analysis in the CircBase database.

## 2 MATERIALS AND METHODS

### 2.1 Subject recruitment

In total, 56 patients diagnosed with Kawasaki disease in the Ningbo Women and Children's Hospital from January 2017 to December 2017 were recruited, including 36 males and 20 females. The minimum age of KD onset was 10 months, and the maximum age of onset was 7 years. The average age was 2.58 ± 1.73 years. Additionally, we collected 56 age‐ and gender‐matched children who were physically healthy, without any clinical signs of infection or inflammation as controls. All patients diagnosed with KD had fever for at least five days and met at least four of the five clinical criteria for KD (lips and oral cavity, bilateral conjunctival congestion, acute non‐purulent cervical lymphadenopathy, polymorphous exanthema and changes in the extremities) or three of the five criteria plus coronary artery abnormalities documented by echocardiogram.^13^ The follow‐up of all patients was record clinical and laboratory information, whether there is any abnormal coronary artery by cardiac color ultrasound. The demographic and clinical characteristics of the study subjects are summarized in Table 1. Serum samples were obtained from KD subjects at two points: within 24 hours before intravenous immunoglobulin (IVIG) therapy, followed by at 3 or 4 days after IVIG therapy. Serum samples were obtained from the controls only once. All patients with KD received 2 g/kg IVIG and were administered aspirin orally (Table 2).

**Table 1:** Demographic data

	Healthy control (N = 56)	Patients with Kawasaki disease (N = 56)
Age, y	3.18 ± 3.26	2.58 ± 1.73
Male gender	31 (55.36%)	36 (64.29%)
Clinical data		Average duration of fever:7.37 d
		Changes in the lips and oral cavity changes:52 patients (92.86%)
		Conjunctival congestion:49 patients (87.50%)
		Cervical lymphadenopathy:48 patients (85.71%)
		Polymorphous exanthema:48 patients (85.71%)
		Changes in the extremities:40 patients (71.43%)
		Coronary artery abnormalities:9 patients (16.07%)

**Table 2:** Laboratory data of patients with KD during the acute and convalescent phases

Groups	Acute KD	Convalescent KD	*P*value	Control	*P*value
n	56	56		56	
D‐dimer (μg/L)	1659.643 ± 1470.196	669.411 ± 479.317	<0.001		
PCT (ng/mL)	3.034 ± 7.394	0.079 ± 0.064	0.004		
BNP (pg/mL)	1461.929 ± 1894.599	243.875 ± 374.848	<0.001		
TP (g/L)	63.143 ± 4.820	77.886 ± 7.580	<0.001	67.486 ± 4.102	<0.001
Albumin (g/L)	37.420 ± 3.596	36.527 ± 4.841	0.166	44.802 ± 6.214	<0.001
Sodium (mmol/L)	135.554 ± 2.449	136.196 ± 2.075	0.096	139.661 ± 1.665	<0.001
TG (mmol/L)	1.299 ± 0.652	3.256 ± 9.402	0.125	1.133 ± 0.590	0.159
LDH (U/L)	351.393 ± 91.976	333.357 ± 95.401	0.314	296.536 ± 56.695	<0.001
CK (U/L)	56.643 ± 41.823	73.964 ± 60.373	0.032	152.018 ± 65.905	<0.001
CK‐MB (U/L)	28.639 ± 9.587	33.964 ± 12.375	0.012	31.130 ± 17.646	0.355
ADA (U/L)	20.123 ± 6.130	24.414 ± 6.785	<0.001	15.191 ± 6.390	<0.001
ALT (U/L)	58.036 ± 95.599	26.268 ± 27.650	0.017	14.857 ± 6.940	0.001
AST (U/L)	51.268 ± 104.144	49.584 ± 38.784	0.911	35.5711 ± 9.273	0.264
TC (mmol/L)	3.541 ± 0.672	3.992 ± 0.974	0.001	4.282 ± 0.793	<0.001
LDL (mmol/L)	2.060 ± 0.558	2.882 ± 4.032	0.138	2.248 ± 0.580	0.084
HDL (mmol/L)	0.692 ± 0.229	0.653 ± 0.307	0.378	1.2359 ± 0.298	<0.001
Tbil (µmol/L)	8.721 ± 9.622	4.989 ± 2.534	0.002	5.588 ± 2.589	0.020
Dbil (µmol/L)	3.218 ± 6.100	1.255 ± 0.908	0.012	1.275 ± 0.650	0.020
Ibil (µmol/L)	5.503 ± 4.087	3.676 ± 1.760	<0.001	4.313 ± 2.069	0.054
TBA (µmol/L)	21.873 ± 41.824	5.618 ± 3.622	0.004	6.705 ± 3.933	0.008
UA (µmol/L)	216.018 ± 80.789	251.464 ± 94.194	0.006	248.786 ± 52.935	0.013
pre‐albumin (mg/L)	6.412 ± 2.374	19.048 ± 7.977	<0.001	19.873 ± 4.621	<0.001
WBC (10^9^/L)	13.845 ± 3.919	8.463 ± 3.240	<0.001	9.311 ± 2.748	<0.001
Neutrophil (%)	67.186 ± 15.395	35.429 ± 15.944	<0.001	39.446 ± 14.747	<0.001
Monocyte (%)	7.107 ± 3.850	7.536 ± 2.493	0.480	6.554 ± 2.106	0.347
Basophil (%)	0.696 ± 2.335	0.625 ± 0.489	0.825	0.393 ± 0.493	0.343
RBC (10^9^/L)	4.086 ± 0.320	4.102 ± 0.429	0.728	4.609 ± 0.379	<0.001
Hb (g/dL)	10.614 ± 1.591	10.723 ± 0.996	0.647	12.013 ± 1.135	<0.001
MCHC (g/dL)	32.689 ± 1.320	32.296 ± 2.465	0.196	32.445 ± 1.277	0.321
Platelet count (10^9^/L)	352.625 ± 113.745	571.411 ± 161.416	0.023	319.089 ± 75.504	0.069
PDW (10GSD)	9.727 ± 0.929	9.062 ± 1.341	<0.001	9.220 ± 1.821	0.825
CRP (mg/L)	78.19 ± 53.992	14.632 ± 17.823	0.004	3.821 ± 3.009	<0.001

### 2.2 Collection of human blood samples and RNA extraction

Peripheral blood (3 mL) was collected from a direct venous puncture into a tube containing sodium citrate. Then, whole blood was centrifuged to obtain serum within 48 hours and stored at −80°C until use. RNA was isolated using Trizol‐ls (Qiagen, Hilden, Germany), followed by phenol and chloroform.

### 2.3 Quantitative reverse transcription‐polymerase chain reaction (qRT‐PCR)

RNAs were reverse transcribed with the HiFi‐MMLV cDNA first strand synthesis kit (CWBIO). qRT‐PCRs were performed on a 96‐well format Roche LightCycler 480 real‐time PCR machine to compare the levels of the two circRNAs between the case and control groups. The relative fold changes were calculated by the comparative threshold cycle method, and GAPDH was used as the internal normalization control. Primers are listed in Table 3. The dissociation curve of each sample was then assessed. CircRNA levels were calculated by the ΔCt method with GAPDH as the control. Larger ΔCt values indicate lower expression. All data in this study were expressed as the mean ± standard deviation (SD) of two independent experiments.

**Table 3:** Sequences of primers

Gene name	Primer sequence	Annealing temperature, °C
ANRIL	Sense: 5′‐TGTACTTAACCACTGGACTACCTGCC‐3′	59
	Anti‐sense: 5′‐TCCACCACACCTAACAGTGATGCTTG‐3′	
hsa_circ_0123996	Sense: 5′‐ACACGTGGCATGATCACACA‐3′	59
	Anti‐sense: 5′‐ACTCCTGGACTGGGCTTGAC‐3′	
U6	Sense: 5′‐GGTGAAGCAGGCGTCGGAGG‐3′	59
	Anti‐sense:5′‐ GAGGGCAATGCCAGCCCCAG‐3′	

### 2.4 Statistical analyses

The data were analyzed using SPSS version 24.0 (SPSS, Chicago, IL, USA). Categorical data were presented as count and percentile. Continuous variables were described as means ± SD. Comparisons of KD patients and healthy children were performed using independent samples *t* test. The differences in levels between acute phase and after IVIG therapy were assessed using the *t* test for paired data. Pearson correlation test was used to test correlations between levels of different circRNAs and between circRNAs levels and values from clinical laboratory testing performed on the same serum samples. Receiver operating characteristic (ROC) curves were established to evaluate the diagnostic values of the circRNAs. For all results, *P* < 0.05 was considered statistically significant.

### 2.5 Ethics statement

All patients were enrolled at the Ningbo Women and Children's hospital after obtaining written parental informed consent. The present study was approved by the Ethics Committee of Ningbo Women and Children's hospital.

## 3 RESULTS

### 3.1 Demographic data

Serum samples were collected from 56 KD patients (2.58 ± 1.73 years old, 36 male) in this study, and 56 healthy children (3.18 ± 3.26 years old, 31 male) were chosen as control. There was no significant difference in age or gender between patients with KD and the control groups. Symptoms other than fever included changes in lips and oral cavity (52 cases, 92.9%), bilateral conjunctival congestion (49 cases, 87.5%), acute non‐purulent cervical lymphadenopathy (48 cases, 85.7%), polymorphous exanthema (48 cases, 85.7%) and changes in the extremities (40 cases, 71.4%). Nine patients (16%) were found to have coronary artery abnormalities (Table 1).

All patients with KD received 2 g/kg IVIG and were administered aspirin orally. As shown in Table 2, in acute KD (ie, before therapy) and convalescent KD (ie, after therapy), laboratory parameters of D‐dimer (*P* < 0.001), PCT (*P* = 0.004), BNP (*P* < 0.001), TP (*P* < 0.001), CK (9 = 0.032), CK‐MB (*P* = 0.012), ADA (*P* < 0.001), ALT (*P* = 0.017), TC (*P* = 0.001), Tbil (*P* = 0.002), Dbil (*P* = 0.012), Ibil (*P* < 0.001), TBA (*P* = 0.004), UA (*P* < 0.006), pre‐albumin (*P* < 0.001), WBC (*P* < 0.001), neutrophil count (*P* < 0.001), platelet count (*P* = 0.023), PDW (*P* < 0.001) and CRP (*P* = 0.004) were differed significantly. In addition, the laboratory data of the two groups on admission were not significantly different with regards to albumin level, sodium, TG, LDH, AST, LDL, HDL, monocyte count, basophil count, Hb or MCHC.

However, between patients in the acute KD phase and controls, TP (*P* < 0.001), albumin (*P* < 0.001), sodium (*P* < 0.001), LDH (*P* < 0.001), CK (*P* < 0.001), ADA (*P* < 0.001), ALT (*P* = 0.001), TC (*P* < 0.001), HDL (*P* < 0.001), Tbil (*P* = 0.02), Dbil (*P* = 0.02), TBA (*P* = 0.008), UA (*P* = 0.013), pre‐albumin (*P* < 0.001), WBC (*P* < 0.001), neutrophil count (*P* < 0.001), RBC (*P* < 0.001), Hb (*P* < 0.001) and CRP (*P* < 0.001) were differed significantly. In contrast, TG, CK‐MB, AST, LDL, Ibil, monocyte count, basophil count, MCHC, platelet count, and PDW showed no difference between the two groups.

### 3.2 Expression of circANRIL in KD patients serum

**Figure 1:**
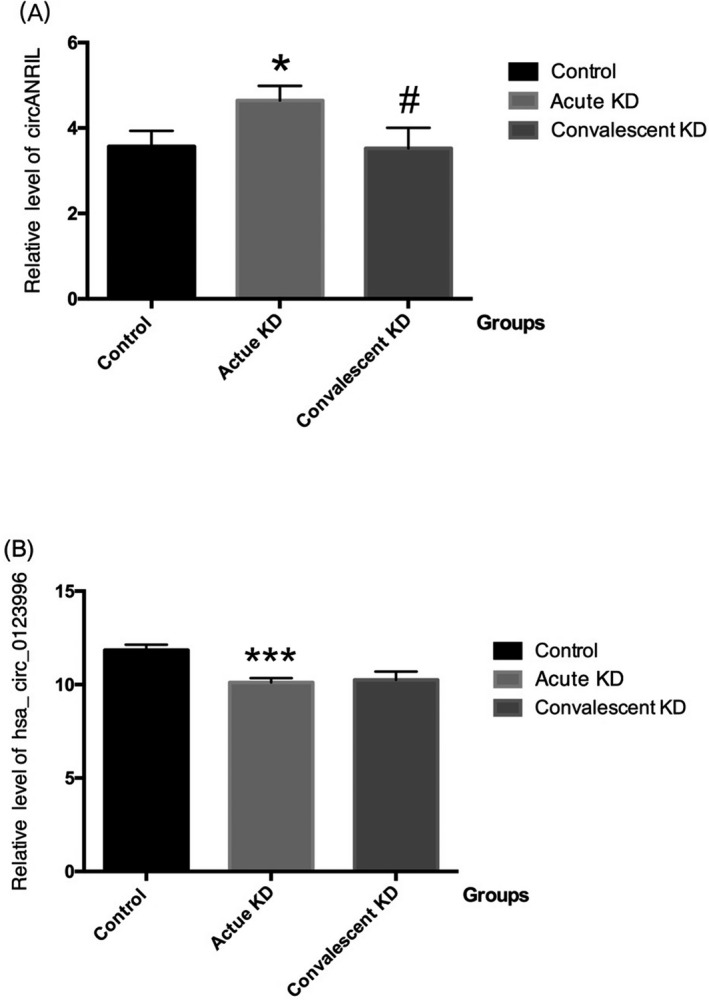
Relative levels of circANRIL and hsa_circ_0123996 in KD patients and controls. (A) Determined the expression of circANRIL by qRT‐PCR. (B) Determined the expression of hsa_circ_0123996 by qRT‐PCR. Data are expressed as mean ± SD. *P < 0.05 control compared with acute KD group; #*P* < 0.05 acute KD group compared with convalescent KD group; ***P < 0.001 control compared with acute KD group

Serum circANRIL level was significantly lower in patients during the acute phase of KD than in controls (*P* = 0.035; Figure 1A). The serum circANRIL level in patients with KD was significantly increased following the IVIG therapy (*P* = 0.016, Figure 1A), to a level that was comparable to the acute phase.

### 3.3 Expression of hsa_circ_0123996 in the serum of patients with KD

The serum level of hsa_circ_0123996 was measured in 45 KD patients during the acute phase and in 45 healthy individuals. The serum hsa_circ_0123996 level was significantly higher in patients during the acute phase of KD than in the controls (*P* < 0.001; Figure 1B). The serum hsa_circ_0123996 level did not change in patients with KD following the IVIG therapy.

### 3.4 Potential diagnostic values of circANRIL and hsa_circ_0123996 for KD

**Figure 2:**
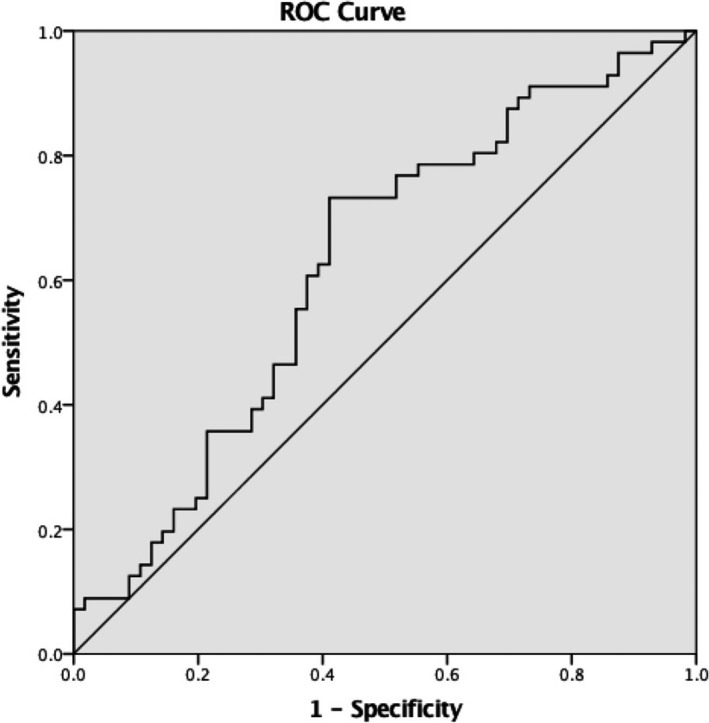
The ROC curve for evaluating the diagnostic value of circANRIL for KD. The area under ROC curve (AUC) was 0.624 (95% CI = 0.520‐0.727). The sensitivity and specificity were 72.3% and 58.9%, respectively

**Figure 3:**
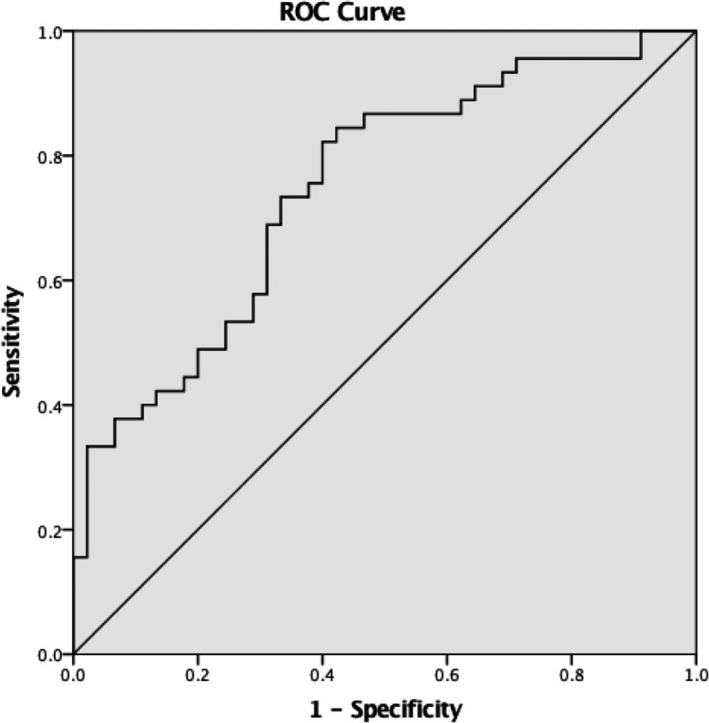
The ROC curve for evaluating the diagnostic value of hsa_circ_0123996 for KD. The area under ROC curve (AUC) was 0.747(95% CI = 0.647‐0.848). The sensitivity and specificity were 82.2% and 60.0%, respectively

To estimate the diagnostic valued of circANRIL and hsa_circ_0123996 for KD, ROC curves were used. The larger the area under the ROC curve (AUC), the higher the diagnostic value. The AUC of circANRIL was 0.624 (95% confidence interval (CI) = 0.520‐0.727; Figure 2). The sensitivity and specificity of circANRIL were up to 72.3% and 58.9%, respectively. For hsa_circ_0123996, AUC was 0.747 (95% CI = 0.647‐0.848; Figure 3). The sensitivity and specificity of hsa_circ_0123996 were up to 82.2% and 60.0%, respectively.

### 3.5 Relationship between circANRIL levels and clinic laboratory factors

To further evaluate the usefulness of circANRIL as KD biomarkers, we tested whether its level correlated with laboratory parameters. As shown in Table 4, serum levels of circANRIL were negatively correlated with albumin level (*r* = −0.197, *P* = 0.037), CK (*r* = −0.217, *P* = 0.022), and pre‐albumin (*r* = −0.211, *P* = 0.026) and positively correlated with CRP (*r* = 0.196, *P* = 0.039), but not associated with other important laboratory features, including TP, sodium, ADA, TC, HDL, Tbil, Dbil, TBA and neutrophil count.

**Table 4:** Correlation of circANRIL and hsa_circ_0123996 level expression with laboratory parameters

Parameter	circANRIL		hsa_circ_0123996	
	Pearson Correlation	*P* value	Pearson Correlation	*P* value
TP (g/L)	−0.056	0.560	0.251	0.017
Albumin (g/L)	−0.197	0.037	0.324	0.002
Sodium (mmol/L)	−0.174	0.066	0.339	0.001
TG (mmol/L)	−0.066	0.488	−0.016	0.882
LDH (U/L)	0.007	0.945	−0.145	0.174
CK (U/L)	−0.217	0.022	0.309	0.003
CK‐MB (U/L)	0.067	0.482	−0.047	0.658
ADA (U/L)	−0.016	0.863	−0.040	0.708
ALT (U/L)	−0.051	0.594	−0.212	0.045
AST (U/L)	−0.052	0.583	−0.099	0.351
TC (mmol/L)	−0.100	0.292	0.288	0.006
LDL (mmol/L)	−0.052	0.583	0.150	0.158
HDL (mmol/L)	−0.115	0.229	0.288	0.006
Tbil (µmol/L)	−0.072	0.451	0.033	0.760
Dbil (µmol/L)	−0.059	0.537	−0.008	0.938
Ibil (µmol/L)	−0.078	0.412	0.085	0.426
TBA (µmol/L)	−0.071	0.455	−0.092	0.391
UA (µmol/L)	−0.030	0.751	0.357	0.001
pre‐albumin (mg/L)	−0.211	0.026	0.430	<0.001
WBC (10^9^/L)	0.055	0.566	−0.178	0.094
Neutrophil (%)	0.143	0.133	−0.159	0.134
Monocyte (%)	0.236	0.012	−0.156	0.142
Basophil (%)	−0.277	0.003	0.025	0.813
RBC (10^9^/L)	−0.029	0.761	0.282	0.007
Hb (g/dl)	−0.028	0.771	0.081	0.445
MCHC (g/dL)	0.095	0.318	−0.370	<0.001
Platelet count (10^9^/L)	0.036	0.704	0.081	0.447
PDW (10GSD)	−0.157	0.098	−0.106	0.319
CRP (mg/L)	0.196	0.039	−0.279	0.008

A multivariable linear regression analysis showed that basophil count, CK, monocyte count and ADA had impacts on the circANRIL level (Table 5). The mathematic model of circANRIL from the multivariable linear regression analysis was *y* = 5.536 − 0.479*x*_1_−0.011*x*_2 _+ 0.199*x*_3 _− 0.081*x*_4_.

**Table 5:** CircANRIL level expression between acute KD and healthy children by the multivariable linear regression

Parameter	*B*	SE	*T*	*P*	95% (lower, upper)
Basophil (*X*_1_)	−0.479	0.141	−3.397	0.001	(−0.759, −0.200)
CK (*X*_2_)	−0.011	0.003	−3.128	0.002	(−0.017, −0.004)
Monocyte (*X*_3_)	0.199	0.079	2.537	0.013	(0.044, 0.355)
ADA (*X*_4_)	−0.081	0.038	−2.120	0.036	(−0.156, 0.005)
Constant	5.536	0.966	5.534	<0.001	(3.621, 7.450)

### 3.6 Association between hsa_circ_0123996 expression and clinical laboratory factors in KD

Laboratory parameters of 45 serum samples from KD patients and 56 control serum samples were analyzed. The data summarized in Table 4 show that hsa_circ_0123996 level was positively correlated with TP (*r* = 0.251, *P* = 0.017), albumin (*r* = 0.324, *P* = 0.002), sodium (*r* = 0.339, *P* = 0.001), UA (*r* = 0.357, *P* = 0.001), TC (*r* = 0.288, *P* = 0.006), HDL (*r* = 0.288, *P* = 0.006), CK‐MB (*r* = 0.309, *P* = 0.003) and pre‐albumin (*r* = 0.430, *P* < 0.001), whereas hsa_circ_0123996 level negatively correlated with ALT (*r* = −0.212, *P* = 0.045) and CRP (*r* = −0.279, *P* = 0.008).

By using multivariable linear regression, pre‐albumin, MCHC, UA, PDW and Ibil had influence on hsa_circ_0123996 levels (Table 6). The mathematic model of hsa_circ_0123996 from the multivariable linear regression analysis was *y* = 30.045 + 0.078*x*_1 _− 0.545*x*_2 _+ 0.006*x*_3 _− 0.429*x*_4 _+ 0.124*x*_5_).

**Table 6:** Hsa_circ_0123996 level expression between acute KD and healthy children by the multivariable linear regression

Parameter	*B*	SE	*T*	*P*	95% (lower, upper)
Pre‐albumin (*X*_1_)	0.078	0.025	3.163	0.002	(0.029, 0.126)
MCHC (*X*_2_)	−0.545	0.148	−3.678	<0.001	(−0.84, −0.251)
UA (*X*_3_)	0.006	0.002	2.253	0.027	(0.001, 0.011)
PDW (*X*_4_)	−0.429	0.160	−2.691	0.009	(−0.747, −0.112)
Ibil (*X*_5_)	0.124	0.051	2.461	0.016	(0.024, −0.225)
Constant	30.045	5.429	5.534	<0.001	(19.248, 40.841)

## 4 DISCUSSION

Kawasaki disease is an acute vasculitis syndrome in infants and young children that affects small‐ and medium‐sized arteries, particularly the coronary arteries.^14^ The cardiovascular problems in KD mainly involve coronary artery lesions, which may result in aneurysm formation, thrombotic occlusion, progression to coronary artery disease and premature atherosclerosis.^15^ Sugimura et al^16^ discovered marked thickening of the intima and calcification in the coronary aneurysms after KD in an intravascular ultrasound study of the coronary artery. These lesions are similar to arteriosclerotic lesions. Suzuki et al^17^ observed active expression of various growth factors in the coronary arteries during the late phase of KD, suggesting that active vascular remodeling continues even several years after the onset. Takahashi et al^18^ reported that the coronary arteries in adults who had suffered KD in their childhood demonstrate premature atherosclerotic changes. These results suggest that coronary arteries in patient who have had KD may be at increased risk of atherosclerotic processes. Currently, there are no specific tests or biomarkers for early diagnosis of KD. CircANRIL is a circular anti‐sense non‐coding RNA near the INK4/ARF locus, which is transcribed from a locus of atherosclerotic cardiovascular disease on chromosome 9p21.^5^ The expression of circANRIL correlates with atherosclerotic vascular disease risk.^19^ Recently, Lesca M. Holdt et al suggested that circANRIL is a potential therapeutic target for the treatment of atherosclerosis, since it might promote antiatherogenic cell functions and is particularly stable against degradation.^5^ Here, we show that the serum circANRIL level was significantly lower in patients during the acute phase of KD than in controls. Interestingly, the serum circANRIL level increases in KD patients after the IVIG therapy (convalescent KD).It is well established IVIG administered in the acute phase of KD reduces the prevalence of coronary artery abnormalities. A meta‐analysis of IVIG compared with placebo has conclusively shown a decrease in new coronary artery abnormalities in IVIG‐treated patients.^20^ However, the mechanism of IVIG in the treatment of KD is unknown; circANRIL may play a profound role in the efficacy of IVIG in the treatment of KD. CircANRIL might serve as a novel potential biomarker for KD and further experiments are needed to validate its usefulness. A larger cohort should be analyzed in the future to test the specificity and sensitivity of using circANRIL to diagnose KD.

According to CircBase database, hsa_circ_0123996 is a circRNAs with 1009nt in spliced sequence length. Its gene is located at chr3:48416843‐48423575 in the human genome, and its associated gene symbol is FBXW12. Due to the limitation of serum samples quantity, we only collected 45 serum samples from KD patients. In this study, the serum hsa_circ_0123996 level was found to be significantly higher in patients with KD than in the controls; however, its level did not change in KD patients following therapy. For hsa_ circ_0123996, the AUC was 0.747. The current finding suggests that hsa_circ_0123996 has an important role in the pathophysiology of KD, and the serum hsa_circ_0123996 level might serve as a new biomarker for early KD diagnosis.

Although KD diagnosis is clinical, laboratory findings (elevated erythrocyte sedimentation rate and C‐reactive protein level, hyponatremia, hypoalbuminemia and elevated liver enzyme levels) may be helpful in evaluating suspected cases and differentiating KD from other conditions.^19^ We analyzed a number of laboratory parameters and found that D‐dimer, PCT, BNP, TP, CK‐MB, ADA, ALT, TC, Tbil, Dbil, Ibil, TBA, UA, pre‐albumin, WBC, neutrophil count, platelet count, PDW and CRP differed significantly between acute KD and convalescent KD. Additionally, between patients with acute KD and controls, TP, albumin, sodium, LDH, CK, ADA, ALT, TC, HDL, Tbil, Dbil, TBA, pre‐albumin, WBC, neutrophil count and CRP differed significantly. These differences are mainly in inflammation indicators, liver and kidney functions and blood lipid levels. Therefore, KD affects a variety of laboratory indicators and is associated with multiple organ damage.

Furthermore, we analyzed the relationship between this circular RNA levels and clinical laboratory parameters in KD patients and healthy individuals. We found that the serum circANRIL level was negatively correlated with albumin level, CK, and pre‐albumin and positively correlated with CRP. A multivariable linear regression analysis showed that basophil, CK, monocyte count and ADA had impacts on the serum circANRIL level. In addition, the serum hsa_circ_0123996 level was positively correlated with TP, albumin, sodium, UA, TC, HDL, CK‐MB and pre‐albumin but negatively correlated with ALT and CRP. Pre‐albumin, MCHC, UA, PDW and Ibil had influence on the serum hsa_circ_0123996 level as indicated by a multivariable linear regression analysis. Thus, the serum levels of circANRIL and hsa_circ_0123996 in KD are related to a variety of laboratory indicators in KD. More experiments are needed to investigate the role of circANRIL and hsa_circ_0123996 in KD.

In conclusion, our study showed that the serum circANRIL level was lower in serum patients during the acute phase of KD than in controls and was increased in the patients following IVIG. The serum hsa_circ_0123996 level was higher in KD patients than in healthy controls. Our results suggest that circANRIL and hsa_circ_0123996 may serve as novel biomarkers for the diagnosis of KD.
